# Editorial: Ocular surface disorders- an insight

**DOI:** 10.3389/fopht.2026.1895030

**Published:** 2026-06-26

**Authors:** Hon Shing Ong, Shweta Agarwal

**Affiliations:** 1 Corneal and External Diseases Department, Singapore National Eye Centre, Singapore, Singapore; 2 Regenerative Therapy Research Group, Singapore Eye Research Institute, Singapore, Singapore; 3 Department of Ophthalmology and Visual Science, Duke-NUS Medical School, Singapore, Singapore; 4 CJ Shah Cornea Services, Medical Research Foundation, Sankara Nethralaya, Chennai, Tamil Nadu, India

**Keywords:** dysbiosis, exposome, immunology, immunometabolism, inflammation, microbiome, ocular surface

The ocular surface is increasingly recognized as a dynamic ecosystem in which microbial communities, immune responses, and environmental exposures interact continuously. This integrated perspective is particularly relevant to ocular surface disorders, where disease often reflects disruption of this finely balanced system rather than a single isolated factor (1).

The ocular surface microbiome (OSM) comprises a community of microorganisms—primarily bacteria, along with fungi and viruses—that colonize the ocular surface and contribute to its homeostasis. Commensal microbes play important roles in maintaining immune tolerance, supporting epithelial barrier integrity, and regulating local immune responses (2). As with other body sites, the microbial composition of the ocular surface is site-specific, and changes may contribute to disease. A healthy microbiome is generally balanced, whereas dysbiosis can disturb homeostasis and promote pathogenic behaviour.

The ocular surface is a low-biomass environment compared to other mucosal sites such as the gut, yet it harbours a distinct and functionally relevant microbial community. Traditional culture-based studies have shown variable detection rates, most commonly identifying coagulase-negative *Staphylococcus* and *Corynebacterium* species. More recently, next-generation sequencing techniques have improved detection sensitivity but have also introduced challenges related to contamination, sampling variability, and data interpretation, leading to inconsistent findings across studies (2, 3).

Within this ecosystem, the OSM plays a key role in maintaining immune balance. During chronic inflammation, disruption of the epithelial barrier can alter host–microbe interactions, allowing normally commensal organisms to behave more like pathogens. This has been observed in severe conditions such as Stevens–Johnson syndrome, where microbial imbalance and epithelial damage contribute to persistent inflammation and recurrent infections (2). Although a clearly defined “core” microbiome is still lacking, commonly identified genera such as *Corynebacterium*, *Staphylococcus*, and *Streptococcus* suggest shared roles in maintaining ocular surface homeostasis (2). Microbial imbalance has been associated with several ocular surface diseases. In dry eye disease, alterations in microbial composition may increase inflammation and contribute to tear film instability. In meibomian gland dysfunction, changes in bacterial populations—particularly increased *Staphylococcus* species—can affect lipid composition through lipase activity, further destabilizing the tear film. In allergic eye disease (e.g. atopic keratoconjunctivitis, vernal keratoconjunctivitis), the microbiome may interact with an already dysregulated immune system, worsening inflammation and barrier dysfunction (3). However, findings across studies remain variable, highlighting the need for standardized methods and larger datasets.

Beyond the ocular surface, the gut microbiome also influences ocular health through the gut–eye axis. Changes in gut microbial composition can affect systemic immune responses that extend to the ocular surface. Gut dysbiosis has been linked to conditions such as Sjögren’s syndrome and dry eye disease, suggesting that microbial products and immune signals from the gut may contribute to ocular surface inflammation (3, 4). These observations emphasize the importance of considering both local and systemic microbial interactions.

Parallel to microbial influences, the immune architecture of the ocular surface is highly specialized and tightly regulated. It serves as an active immunological interface that responds to environmental and endogenous signals while maintaining tolerance. Disruption of this balance contributes to a range of ocular surface diseases with distinct immune mechanisms (5). Autoimmune conditions such as Sjögren’s syndrome involve immune-mediated damage to the lacrimal glands, leading to tear deficiency and chronic inflammation. Chronic allergic diseases are driven mainly by Th2 responses, whereas dry eye disease is more often associated with Th1 and Th17 pathways. More severe conditions, such as cicatrizing conjunctivitis, peripheral ulcerative keratitis and Mooren’s ulcer, reflect intense immune activation and tissue destruction (5, 6). These differences highlight that ocular surface disease is driven by multiple, context-specific immune pathways rather than a single mechanism (7).

Immunometabolism adds another layer to this complexity. Metabolic pathways within immune cells influence their activation and function, linking cellular energy states to inflammatory responses. Tear metabolomic studies support this connection, showing that metabolic changes at the ocular surface are closely associated with immune activity. Within the lacrimal functional unit—which integrates the cornea, conjunctiva, lacrimal glands, and their neural and immune connections—the ocular surface functions as a coordinated system (8, 9). The interaction between microbial signals, immune responses, and metabolic regulation is essential for maintaining epithelial integrity and visual function. When this balance is disrupted chronic inflammation becomes a major driver of tissue damage (9).

The environmental dimension of this ecosystem is equally important, as the ocular surface is continuously exposed to external influences. Allergens, pollutants, and climatic conditions not only trigger symptoms but also shape immune responses. In ocular allergy, early subclinical changes suggest that environmental exposures may initiate immune activation before clinical signs become evident (10). Climatic factors such as high temperature, low humidity, and wind increase tear evaporation, leading to tear film instability and worsening of dry eye symptoms. Sunlight and ultraviolet exposure can further aggravate inflammation, while dust and airborne allergens are common triggers for allergic disease. Air pollution has emerged as a significant contributor to ocular surface morbidity, particularly in individuals with underlying immune susceptibility (10, 11). Although inflammatory pathways are implicated, the exact mechanisms by which pollutants affect ocular surface tissues are not yet fully understood. The concept of the exposome provides a useful framework to integrate these influences. External exosomes include environmental and lifestyle factors such as pollution, climate, and stress, while internal exposomes include the microbiome and oxidative stress (11). These factors interact with the host genome through epigenetic changes, altering gene expression and influencing disease susceptibility. At the cellular level, the ocular surface epithelium acts as an active immune interface. Pattern recognition receptors, including Toll-like and NOD-like receptors, detect environmental stimuli and activate signaling pathways such as NF-κB and MAPK. This leads to the release of inflammatory cytokines and chemokines, linking innate and adaptive immune responses. Through these mechanisms, environmental exposures directly influence immune activity and epithelial health (11, 12).

Despite growing advances, several challenges remain in fully understanding the ocular surface as an integrated ecosystem. Variability in microbiome studies, lack of standardized sampling and analytical methods, and the low biomass nature of the ocular surface continue to limit reproducibility and interpretation. Similarly, the complex and context-specific nature of immune responses, along with emerging fields such as immunometabolism and exposomics, requires further validation through well-designed longitudinal and translational studies. Integrating multi-omics data with clinical phenotypes and environmental exposures also remains a key unmet need.

**Figure 1:**
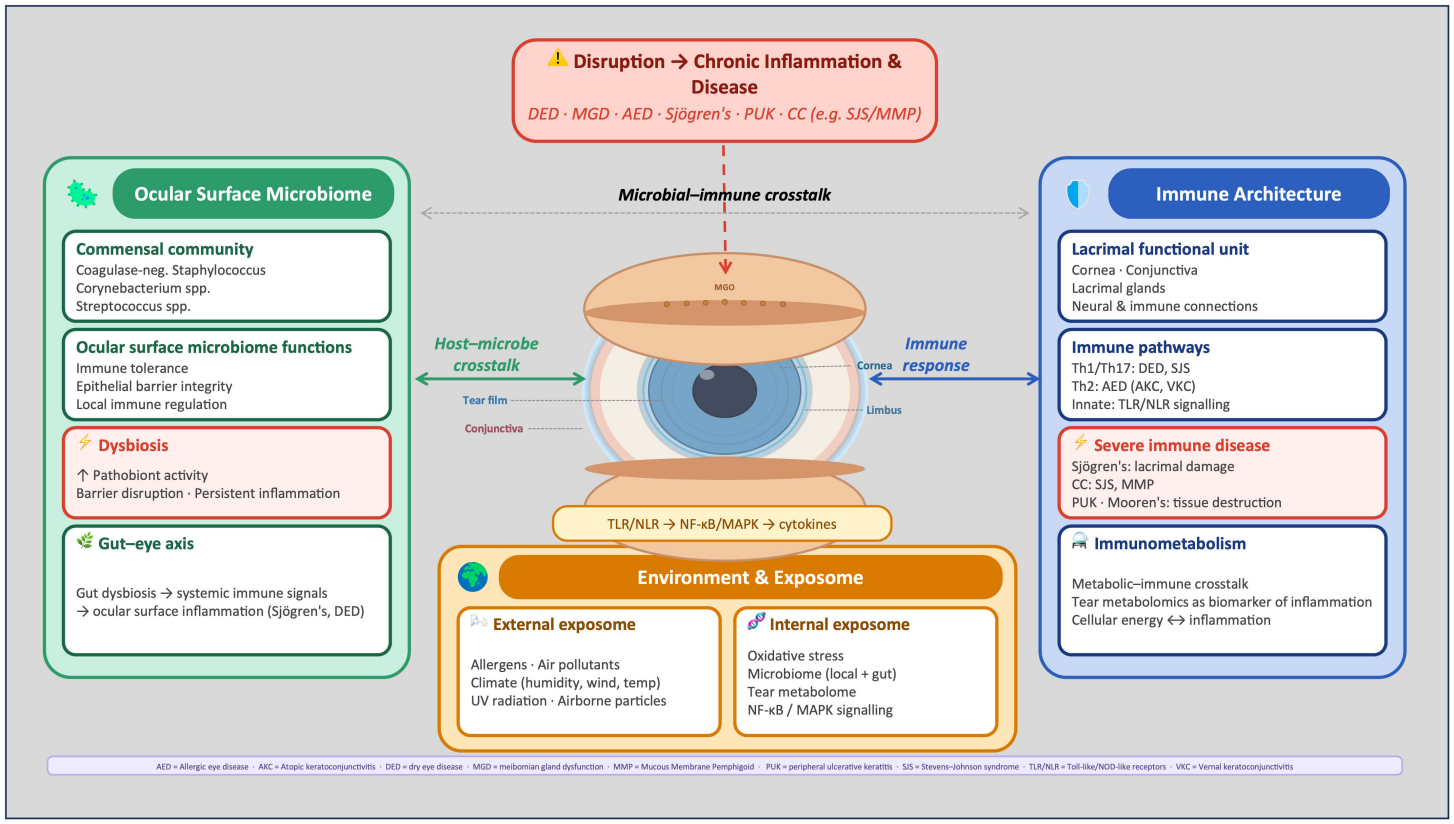
The ocular surface as an integrated ecosystem.

In summary, the ocular surface should be viewed as an integrated and dynamic ecosystem, where microbiome, immunity, and environment are closely interconnected. (**Figure 1**) Disruption at any level of this network can shift the system toward chronic inflammation and disease. A better understanding of these interactions will be essential for developing future therapies that aim not only to control symptoms but to restore and maintain ocular surface homeostasis.
